# Effect of chemically protected sodium butyrate on growth performance, serum indicators, rumen fermentation parameters, and microbiota of Gangba sheep

**DOI:** 10.5713/ab.250681

**Published:** 2026-03-11

**Authors:** Yining Xie, Mebratu Melaku, Zhaohan Zhan, Xiaokang Jing, Junhong Wang, Huaibao Zhao, Bao Yi, Teng Ma, Liang Chen, Hongfu Zhang

**Affiliations:** 1 State Key Laboratory of Animal Nutrition and Feeding, Key Laboratory of Animal Nutrition and Feed Science of Ministry of Agriculture and Rural Affairs, Institute of Animal Science, Chinese Academy of Agricultural Sciences, Beijing, China; 2 Precision Livestock and Nutrition Unit, Gembloux Agro-Bio Tech, TERRA Teaching and Research Centre, Liège University, Gembloux, Belgium

**Keywords:** Chemically Protected Sodium Butyrate, Gangba Sheep, Growth Performance, Rumen Fermentation Parameters, Rumen Microbiota, Serum Indicators

## Abstract

**Objective:**

This study investigated the effects of chemically protected sodium butyrate (CSB) on growth performance, serum indicators, rumen fermentation parameters, and microbial communities in Gangba sheep.

**Methods:**

Twenty-four healthy 5-month-old male Gangba sheep with an initial body weight of 19.54±1.04 kg were randomly assigned to four groups and fed diets containing 0, 1.0, 5.0, and 10.0 g/kg CSB for 74 days. After a 14-day adaptation period, the daily feed intake of Gangba sheep was measured. Fecal samples were collected during the last 7 days of the trial, and serum and rumen fluid were sampled on the final day.

**Results:**

CSB improved final body weight, average daily dry matter intake, and average daily gain while reducing the feed conversion ratio of Gangba sheep (p<0.05). In addition, CSB improved the apparent digestibility of crude protein, dry matter, and neutral detergent fiber (p<0.05). Furthermore, CSB increased serum immunoglobulin G levels while decreasing tumor necrosis factor-α levels (p<0.05). Meanwhile, CSB increased serum growth hormone-releasing hormone levels (p<0.05). Furthermore, CSB increased rumen fluid pH and concentrations of ammonia nitrogen, acetic acid, iso-butyric acid, butyric acid, and total volatile fatty acids, as well as activities of lipase and butyrate kinase (p<0.05). Besides, CSB enhanced the rumen microbiota structure by increasing the relative abundance of *Fibrobacter*, *unclassified Oscillospiraceae*, and *Ruminococcus*.

**Conclusion:**

CSB improved immunological and hormone indicators, enhanced the rumen ecological environment, and increased feed digestibility and utilization, thereby promoting the growth of Gangba sheep. It is recommended to use a dosage of 5.0 g/kg dry matter for optimal growth performance and health benefits.

## INTRODUCTION

With the increasing global demand for high-quality meat, rapid growth ate and feed efficiency has become a primary goal in ruminant production systems [1]. In the Qinghai-Tibet Plateau (QTP) region, seasonal market demands for mutton during peak tourist seasons drive herders to intensify feeding, often by supplementing local sheep like the Tibetan sheep with concentrated feeds [2]. However, this practice is challenged by the limited availability and often suboptimal quality of local feed resources, which may compromise the health and growth performance of these plateau-adapted sheep [3].

High-altitude environments impose unique physiological and nutritional challenges on grazing ruminants. Compared to low-altitude animals, ruminants living under chronic hypoxic, low temperature, and low atmospheric pressure exhibit distinct alterations in rumen function, including modified fermentation patterns, reduced fiber degradation efficiency, and reshaped rumen microbial community structures [4,5]. When high-concentrate diets are introduced under these already challenging conditions, the risk of ruminal acidosis and fermentation instability increases further. Therefore, nutritional strategies capable of stabilizing the ruminal environment and optimizing fermentation are particularly critical for sustainable ruminant production in high-altitude regions.

Butyric acid serves as a key energy source for intestinal mucosal cells. It helps maintain intestinal barrier integrity, modulates immune responses, promotes the growth of beneficial intestinal microorganisms, and contributes to improved feed efficiency [6,7]. However, due to its special odor, direct addition of butyric acid to feed would severely impair palatability. Therefore, in practice, butyrate derivatives such as its salts are commonly used instead [8,9]. Previous studies have shown that dietary sodium butyrate supplementation in sheep fed high-concentrate diets can increase the rumen epithelial thickness and upregulates claudin-4 expression, while Clostridium butyricum supplementation can modulate skeletal muscle development and meat quality in lambs by shaping the intestinal microbiota [10,11].

Chemically protected sodium butyrate (CSB) is a composite formulation in which sodium butyrate is encapsulated within a protective matrix consisting of buffering salts—sodium dihydrogen phosphate and sodium bicarbonate [12–14]. This structure allows more efficient release and utilization of butyrate in the gastrointestinal tract. The buffering salts in the matrix help stabilize gastrointestinal pH [15,16], which is critical for maintaining rumen microbial community stability and enzyme activity [17,18]. Studies in monogastric species, such as piglets and broilers, have demonstrated that CSB can enhance intestinal morphology, immune function, and microbial community composition [12–14], indicating its potential as a functional feed additive in ruminant production.

Despite these promising findings, critical knowledge gaps remain. Research on the dose-response effects of CSB in ruminants is scarce, especially under the compounded challenges of high-altitude stress and high-concentrate feeding. However, research on the dose-response effects of CSB in ruminants remains scarce, especially under the compounded challenges of high-altitude stress and high-concentrate feeding. This is especially true for the Gangba sheep, an indigenous breed of the QTP. Reared at altitudes above 4,700 m, this breed has a rumen ecology that is particularly susceptible to dietary and environmental perturbations. The impact of CSB on Gangba sheep is therefore virtually unexplored [3]. While CSB’s buffering capacity and microbiota-modulating properties imply potential benefits, its efficacy in improving an integrated set of outcomes—including growth performance, nutrient digestibility, systemic metabolism, rumen fermentation, and microbial structure—in Gangba sheep remains unknown.

Therefore, we hypothesize that dietary CSB supplementation during concentrate feeding may improve rumen microbial community structure, optimize the ruminal physicochemical environment, and enhance feed utilization efficiency. This study aimed to investigate the effects of graded levels of CSB on growth performance, nutrient digestibility, serum immunological and endocrine indicators, rumen fermentation parameters, enzyme activities, and the rumen microbiota in Gangba sheep raised under high-altitude conditions. The findings are expected to provide a scientific basis for the application of CSB in high-altitude ruminant production systems.

## MATERIALS AND METHODS

### Grouping and treatment of animals

The CSB used was provided by Beijing Shengtaiyuan Biotechnology. It contained 54% sodium butyrate and was protected by a physical and chemical matrix of buffering salts (NaHCO_3_ and NaH_2_PO_4_) [14].

Twenty-four healthy male Gangba sheep, aged 5 months and with an initial body weight of 19.54±1.04 kg, were randomly divided into four treatment groups (n = 6). On a dry matter (DM) basis, the four dietary treatments were as follows: the control diet (CON; Table 1); the low-concentration sodium butyrate diet (CON+1.0 g/kg CSB, LCSB, providing 0.43 g butyrate/kg DM); the medium-concentration sodium butyrate diet (CON+5.0 g/kg CSB, MCSB, providing 2.16 g butyrate/kg DM); and the high-concentration sodium butyrate diet (CON+10.0 g/kg CSB, HCSB, providing 4.32 g butyrate/kg DM). The study took place over a span of 74 days, from July to September 2024, comprising a 14-day pre-experimental phase followed by a 60-day experimental phase. All sheep were housed indoors and individually fed in 1 m×1 m open-mesh stainless-steel pens, each holding one animal. The sheep were fed and provided with water at 08:00 and 17:00 daily, allowing them access to both feed and water freely, while their daily consumption was monitored. Bedding was provided and replaced daily to maintain hygiene and animal comfort. This information was utilized to compute the average daily gain (ADG), dry matter intake (DMI), as well as the feed conversion ratio (FCR).

### Fecal collection, chemical analysis and apparent digestibility of nutrients

During the last 7 days of the experiment, feed and fecal samples were collected from each lamb for nutrient digestibility analysis. Fecal samples were collected twice daily, approximately 2 hours after the morning and afternoon feedings. For each animal, the two daily subsamples were composited in equal proportions to obtain one daily fecal sample. The seven daily composites were then homogenized in equal proportions to produce one final fecal sample per animal. The final fecal and corresponding feed samples were analyzed for crude protein (CP), ether extract (EE), neutral detergent fiber (NDF), acid detergent fiber (ADF), and organic matter (OM). Specially, the analysis methods for feed and fecal samples were as follows: DM, CP, EE, and ash were analyzed according to AOAC methods 934.05, 984.13, 920.39, and 975.03, respectively. NDF and ADF were determined according to the procedures described in previous study [9,19]. The content of OM was calculated using the formula OM (%) = 100–Ash (%).

### Blood and rumen fluid collection

On day 60, blood and rumen samples were collected from all 24 Gangba sheep. Following an overnight fast, blood samples were obtained from the jugular vein using vacuum tubes 2 h before the morning feeding and centrifuged at 3,000×g for 15 minutes to separate the serum. At the same time point, rumen fluid was collected using a hose-type rumen sampler. To minimize saliva contamination, the initial portion to avoid saliva contamination was discarded, following established procedures for oral stomach-tube sampling in small ruminants [20,21]. The sample was filtered through four layers of gauze, and its pH was measured immediately. The remaining rumen fluid was centrifuged at 10,000×g for 15 min at 4°C, and the supernatant was stored at −80°C until further analysis.

### Analysis of serum indicators

Serum immunoglobulins (IgA, IgM, and IgG), cytokines (interleukins IL-2, IL-4, IL-6, and IL-10; tumor necrosis factor-α [TNF-α]; and interferon-γ [IFN-γ]), and endocrine factors (growth hormone [GH], insulin-like growth factor-1 [IGF-1], growth hormone-releasing hormone [GHRH], growth hormone-inhibiting hormone [GHIH], and myostatin) were quantified using sheep-specific ELISA kits according to the manufacturer’s instructions (Shanghai Jining Institute of Bio-Engineering). The kit details are as follows: IgA (Sheep IgA ELISA Kit, Cat. No. JN82299), IgM (Sheep IgM ELISA Kit, Cat. No. JN20997), IgG (Sheep IgG ELISA Kit, Cat. No. JN82597), IL-2 (Sheep IL-2 ELISA Kit, Cat. No. JN22035), IL-4 (Sheep IL-4 ELISA Kit, Cat. No. JN21766), IL-6 (Sheep IL-6 ELISA Kit, Cat. No. JN21752), IL-10 (Sheep IL-10 ELISA Kit, Cat. No. JN83491), TNF-α (Sheep TNF-α ELISA Kit, Cat. No. JN20906), IFN-γ (Sheep IFN-γ ELISA Kit, Cat. No. JN20780), GH (Sheep GH ELISA Kit, Cat. No. JN83713), IGF-1 (Sheep IGF-1 ELISA Kit, Cat. No. JN81107), GHRH (Sheep GHRH ELISA Kit, Cat. No. JN81703), GHIH (Sheep GHIH ELISA Kit, Cat. No. JN82001), and myostatin (Sheep MSTN ELISA Kit, Cat. No. JN80511).

### Analysis of rumen fermentation parameters and enzyme activities

A standard solution A was prepared by mixing 6 volatile fatty acids (VFAs) (acetate acid, propionate acid, butyrate acid, isobutyrate acid, valerate acid, and isovalerate acid) in 10.0 mL of HPLC-grade butanol, and an internal standard solution B was prepared by adding 10 microliters of 2-ethylbutyrate to 10.0 mL of butanol [22,23]. These solutions were diluted into seven working solutions for gas chromatography mass spectrometry (GC-MS) analysis. A 25.0 mL rumen fluid sample was processed by adding 5.0 mL of water containing 0.5% phosphoric acid, followed by freeze-grinding (50 Hz, 3 minutes×2), ultrasonication (10 minutes), and centrifugation (4°C, 13,000×g, 15 minutes). Subsequently, 200 microliters of the supernatant was extracted with 200 microliters of butanol containing 10 micrograms per milliliter of 2-ethylbutyrate, and then vortexed (10 seconds), ultrasonicated (10 minutes), and centrifuged again (4°C, 13,000×g, 5 minutes). The final supernatant obtained from the experimental procedure was carefully transferred into a vial in preparation for GC-MS analysis. The analysis itself was conducted using a state-of-the-art Agilent 8890B-5977B/7000D instrument, which was specifically outfitted with an HP FFAP column. To facilitate the separation of compounds, helium was utilized as the carrier gas. The temperature programming for the analysis commenced at a baseline of 80°C. It then underwent an increase of 20°C/min until it reached 120°C. Following this initial ramp-up, the temperature continued to rise at a more gradual rate of 5°C/min until it reached 160°C, where it was maintained. The final stage involved holding the temperature steady at 220°C for a duration of 3 minutes, which was crucial for ensuring proper analysis of the compounds present. In terms of mass spectrometry conditions, the method employed an electron ionization (EI) ion source, with its temperature configured at 230°C. Additionally, the quadrupole temperature was maintained at a set point of 150°C. Data acquisition was carried out in selected ion monitoring (SIM) mode, which was particularly useful for enhancing the detection of specific ions of interest. To ensure the robustness of this analytical method, a quality control (QC) sample was systematically inserted after every 6 samples. This practice aimed to monitor system stability and ensure measurement repeatability. Furthermore, the relative standard deviation (RSD) of the target compounds was stringently required to remain below 15%. Overall, this methodological framework presents itself as both rational and reliable for the determination of VFAs present in rumen fluid, setting a solid foundation for accurate analytical outcomes. In addition, ruminal ammonia nitrogen concentration was determined using UV–visible spectrophotometry (UV-2450; Shimadzu) with absorbance measured at 620 nm [24,25].

The activities of cellulase, lysozyme, neutral xylanase, pectinase, neutral protease, lipase, α-amylase and butyrate kinase were measured using the microplate reader method, based on the content of reducing sugars and changes in absorbance of the enzymatic hydrolysis products [26]. And the activity of pepsin was measured using sheep-specific ELISA kits according to the manufacturer’s instructions (Shanghai Jining Institute of Bio-Engineering).

### High-throughput sequencing analysis

Genomic DNA was isolated from lamb rumen fluid utilizing the E.Z.N.A.R Soil DNA Kit (Omega Bio-tek), followed by an evaluation of DNA quality through 1% agarose gel electrophoresis [27,28]. The amplification of the high-variable V3-V4 region of the bacterial 16S rRNA gene was accomplished with primers 338F (5’-ACTCCTACGGGAGGCAGCAG-3’) and 806R (5’-GGACTACHVGGGTWTCTAAT-3’). The resulting amplified products were sequenced on the Illumina MiSeq PE300 platform (Illumina). Operational taxonomic units (OTUs) were clustered at a 97% similarity threshold using Uparse (ver. 7.0), and chimeric sequences were eliminated. For taxonomic analysis of the 16S rRNA database, the RDP Classifier (ver. 2.11) was employed, establishing a classification threshold of 0.7.

### Calculation and statistical analysis

The calculation formula for apparent digestibility using acid-insoluble ash (AIA) method is as follows [29,30]:


(1) $$\begin{array}{l} \text{Apparent digestibility }(\%)=\\ 100\times (1-[\text{Nutrient content in feces}\times \text{AIA content in diet}]\\ /[\text{Nutrient content in diet}\times \text{AIA content in feces}]) \end{array}$$

The experimental data were examined utilizing SAS 9.4 (SAS Institute) with the PROC MIXED model. The beneficial effects of CSB supplementation in the diet on Gangba sheep were evaluated by comparing the control group with the CSB treatment groups, as well as by assessing linear and quadratic effects. Additionally, Duncan’s multiple range test was employed to assess the significance of differences among the means of the CSB treatment groups. In this study, differences among the means of the CSB treatment groups were considered significant at p<0.05 and as a trend at 0.05≤p<0.10.

The PROC MIXED model included both random and fixed effects and was expressed as follows:


(2) Yij=μ+Li+Tj+ɛij,

where Yij is the dependent variable, μ is the overall mean, Li is the random effect of individual Gangba sheep (i = 1 to 24), Tj is the fixed effect of CSB supplementation (j = 0, 1.0, 5.0, and 10.0 g/kg), and ɛij is the residual error term.

## RESULTS

### Growth performance

CSB supplementation exerted dose–response effects on the growth performance of Gangba sheep (Table 2). Final body weight increased in both the LCSB and MCSB groups relative to CON (p<0.05), with a significant quadratic effect (p<0.05). DMI and ADG increased, whereas FCR decreased, in response to CSB (p<0.05). Importantly, although LCSB and MCSB improved performance, the HCSB did not provide additional benefits, indicating that further increases above 5 g/kg did not enhance growth, consistent with the quadratic response.

### Apparent digestibility of nutrients

CSB supplementation affected the apparent digestibility of specific nutrients (Table 3). CP and DM digestibility increased significantly in the MCSB group compared with the CON group, with both linear and quadratic dose–response effects detected (p<0.05). In addition, NDF digestibility was significantly higher in the LCSB and MCSB groups compared with CON, with a significant quadratic dose effect (p<0.05).

### Serum immune and hormonal parameters

CSB supplementation affected several serum immune and endocrine indicators in a dose-dependent manner (Table 4). IgG concentration increased in the LCSB and HCSB groups compared with CON, and this response followed a significant linear trend (p<0.05). TNF-α levels declined progressively across all CSB-supplemented groups, also displaying a linear dose–response pattern (p<0.05). IL-2 levels decreased in the LCSB and MCSB groups, showing a significant quadratic response (p<0.05). GHRH concentration increased in the LCSB group with a quadratic effect (p<0.05). Additionally, IL-4 concentrations were reduced in all three CSB groups, exhibiting both linear and quadratic trends (p<0.05).

### Rumen fermentation parameters and enzyme activities

Dietary supplementation with CSB modified rumen fermentation characteristics and enzyme activities in a dose-dependent manner (Table 5). Rumen pH, ammonia-nitrogen, and acetate concentrations were elevated across all CSB supplemented groups, exhibiting both linear and quadratic responses (p<0.05). Iso-butyrate, butyrate, and total VFA concentrations were elevated in the LCSB and MCSB groups, following a quadratic pattern (p<0.05). With respect to enzyme activity, lysozyme activity was reduced in the LCSB and MCSB groups, displaying a quadratic trend (p<0.05). Lipase activity rose in the MCSB and HCSB groups in a linear fashion (p<0.05), whereas butyrate kinase activity was specifically enhanced in the MCSB group and also showed a linear response (p<0.05).

### Rumen microbiota

**Figure 1:**
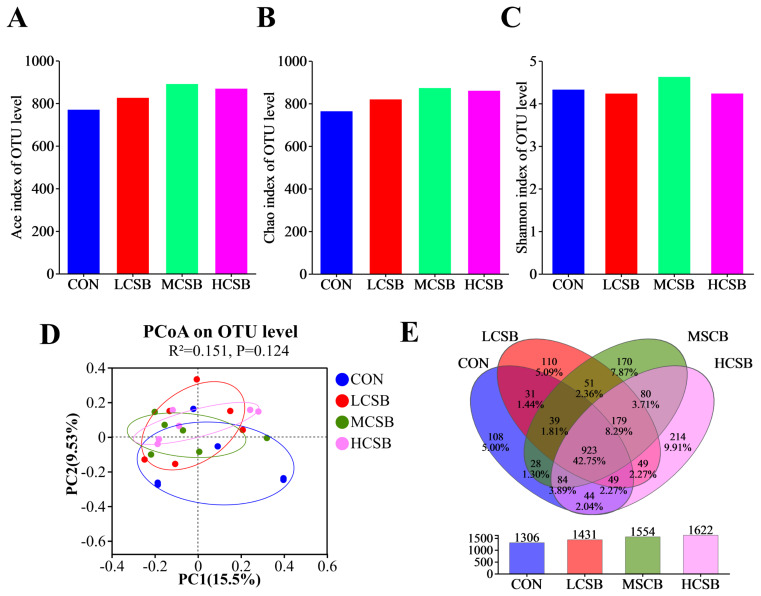
Alpha and beta diversity of rumen microbial communities among treatment groups. (A) Ace index; (B) Chao1 index; (C) Shannon index, representing alpha diversity metrics. (D) Principal coordinates analysis (PCoA) based on Bray–Curtis distances, illustrating beta diversity differences among groups. (E) Venn diagram showing the number of shared and unique operational taxonomic units (OTUs) across treatments. CON: control group; LCSB: low-concentration chemically protected sodium butyrate diet (CON+1.0 g/kg CSB); MCSB: the medium-concentration chemically protected sodium butyrate diet (CON+5.0 g/kg CSB); HCSB: the high-concentration chemically protected sodium butyrate diet (CON+10.0 g/kg CSB).

**Figure 2:**
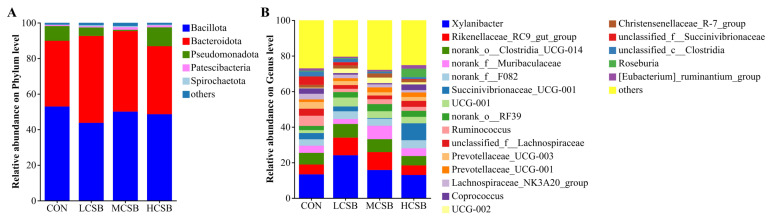
Relative abundance and taxonomic distribution of rumen microbiota across treatment groups. (A) Composition of the rumen microbial community at the phylum level. (B) Composition of the rumen microbial community at the genus level. CON: control group; LCSB: low-concentration chemically protected sodium butyrate diet (CON+1.0 g/kg CSB); MCSB: the medium-concentration chemically protected sodium butyrate diet (CON+5.0 g/kg CSB); HCSB: the high-concentration chemically protected sodium butyrate diet (CON+10.0 g/kg CSB).

**Figure 3:**
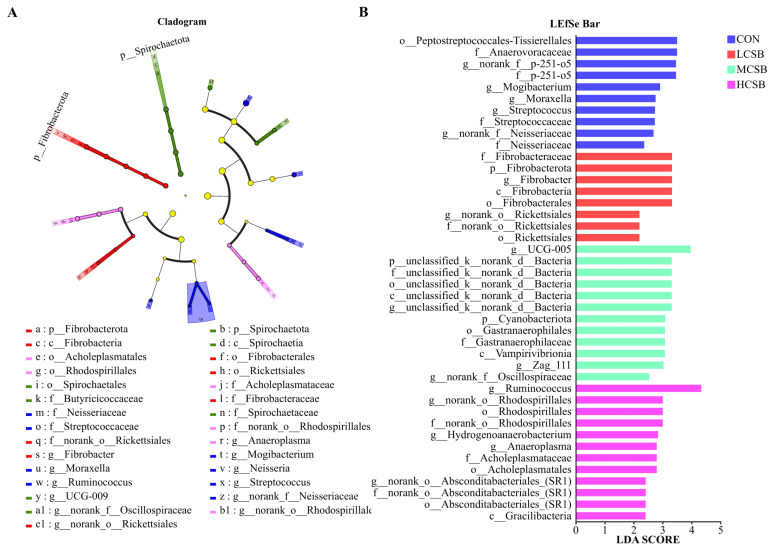
Linear discriminant analysis effect size (LEfSe) analysis of rumen microbial biomarkers. (A) LEfSe cladogram showing the taxonomic distribution of differentially abundant microbial taxa among treatments. (B) LEfSe bar chart displaying the linear discriminant analysis (LDA) scores of taxa with significant differences (LDA score>2), indicating their relative contributions to group discrimination. CON: control group; LCSB: low-concentration chemically protected sodium butyrate diet (CON+1.0 g/kg CSB); MCSB: the medium-concentration chemically protected sodium butyrate diet (CON+5.0 g/kg CSB); HCSB: the high-concentration chemically protected sodium butyrate diet (CON+10.0 g/kg CSB).

In this study, the rumen microbial diversity of 24 Gangba sheep was analyzed, yielding a total of 1,248,254 optimized sequences with 519,125,910 bases, and an average sequence length of 415 bp. Results showed that the addition of CSB had no significant effect on the α diversity (Figures 1A–1C), but tended to change the β diversity (R^2^ = 0.15, p = 0.12, Figure 1D). The Venn diagram (Figure 1E) revealed that the CON group, LCSB group, MCSB group, and HCSB group had 1,306, 1,431, 1,554, and 1,622 OTUs, respectively, with 923 OTUs shared among all four groups, accounting for 42.75%. The bar charts (Figures 2A, 2B) displayed the dominant phyla (top 5) and genera (top 20) of rumen microbes in Gangba sheep at the phylum and genus levels, respectively. The LEfSe analysis revealed that after the addition of CSB, a total of 37 differential microbial biomarkers were identified in the rumen microbiota of Gangba sheep, ranging from the phylum to the genus level (Figures 3A, 3B). Specifically, the CON group had 10 biomarkers, with 5 at the genus level, including *unclassified p-251-o5*, *Mogibacterium*, *Moraxella*, *Streptococcus*, and *unclassified Neisseriaceae*; the LCSB group had 8 biomarkers, with 2 genera identified: *Fibrobacter* and *unclassified Rickettsiales*; the MCSB group had 7 biomarkers, including 3 genera: *UCG-005*, *Zag_111*, and *unclassified Oscillospiraceae*; the HCSB group had 12 biomarkers, with 5 at the genus level: *Ruminococcus*, *unclassified Rhodospirillales*, *Hydrogenoanaerobacterium*, *Anaeroplasma*, and *unclassified Absconditabacteriales_(SR1)*.

### Spearman correlation analysis

**Figure 4:**
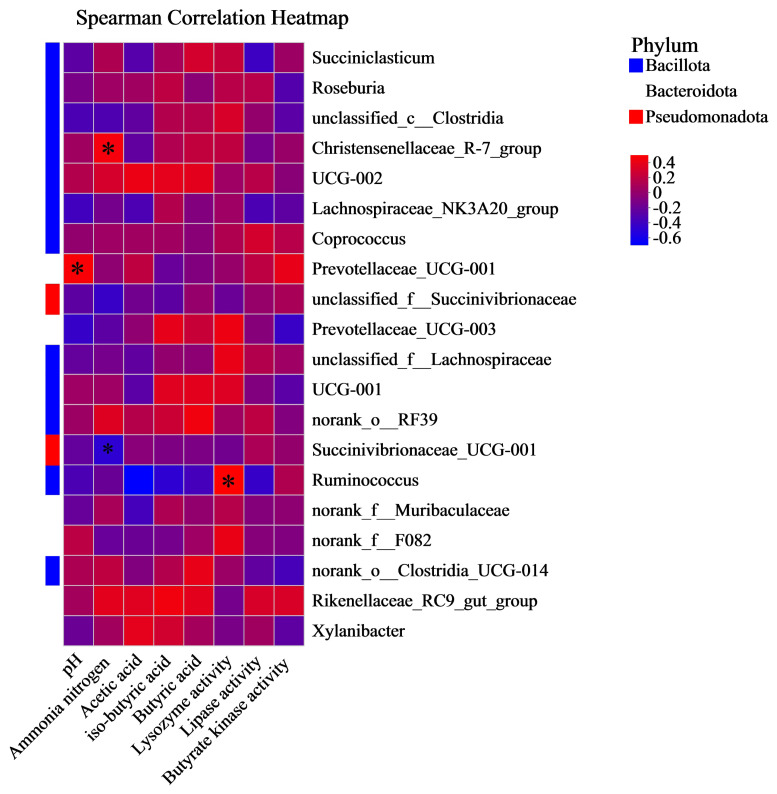
Spearman correlation heatmap. Heatmap showing Spearman’s rank correlations between the differential rumen fermentation parameters and the top 20 rumen microbial taxa (at the genus level). Correlation coefficients (p) are indicated by the color scale, and statistical significance is denoted by asterisks (* p<0.05).

The correlation heatmap results indicated that the value of pH was positively correlated with *Prevotellaceae UCG-001* (R = 0.46, p<0.05, Figure 4). Ammonia nitrogen was positively correlated with *Christensenellaceae R-7_group* (R = 0.41, p<0.05) and negatively correlated with *Succinivibrionaceae* UCG-001 (R = −0.43, p<0.05). Moreover, lysozyme activity was positively correlated with *Ruminococcus* (R = 0.46, p<0.05).

## DISCUSSION

The present study demonstrates that dietary supplementation with CSB elicited significant, dose-dependent improvements in the growth performance of Gangba sheep, as evidenced by increased final body weight, ADG and DMI, alongside a reduced FCR. The most pronounced benefits were observed at the moderate supplementation level of 5.0 g/kg DM (MCSB), with the highest dose (10.0 g/kg DM, HCSB) failing to yield additional advantages, resulting in a clear quadratic dose-response pattern. These performance enhancements are consistent with the well-established role of butyrate as a readily metabolizable energy substrate for intestinal epithelial cells, promoting mucosal integrity, proliferation, and overall nutrient absorption capacity [6,31].

Concurrently, CSB supplementation enhanced systemic immune and endocrine profiles. The observed increase in serum IgG coupled with reductions in the pro-inflammatory cytokines TNF-α, IL-2, and IL-4 aligns with previous reports on the immunomodulatory properties of butyrate, which can enhance humoral immunity while attenuating inflammatory responses [31–33]. Because immune responses are highly specific physiological events, these changes should be interpreted cautiously. Throughout the experimental period, all animals remained clinically healthy with no signs of infection, intestinal injury, or abnormal stress, indicating that the altered immune indices were unlikely to result from pathological stimulation or unintended tissue damage. Instead, these responses more likely reflect improved mucosal barrier integrity and beneficial immunoregulation induced by butyrate supplementation. The elevated concentration of GHRH in CSB-supplemented groups suggests a potential cross-talk between gut-derived signals and the endocrine system, possibly mediated by improved gut barrier function and reduced systemic inflammation affecting the hypothalamic-pituitary axis [34,35]. It must be emphasized, however, that these specific endocrine pathways were not directly measured and remain plausible interpretations derived from indirect evidence.

The improvements in growth were underpinned by enhanced nutrient utilization. CSB supplementation significantly increased the apparent digestibility of CP, NDF and DM corroborating earlier findings in ruminants [36]. These digestibility gains were associated with substantial and favorable restructuring of the rumen microbiota [33]. Moderate CSB supplementation enriched fibrolytic genera such as *Fibrobacter* and *Ruminococcus*, which are fundamental to the degradation of structural plant polysaccharides and contribute significantly to fiber digestion and VFA production [37–39]. *Ruminococcus* also contributes to proteolysis and starch degradation, supporting a broader role in nutrient turnover. These microbial changes likely underlie the enhanced nutrient digestibility observed in the LCSB and MCSB groups.

An integrated analysis reveals that the interconnectedness of microbial ecology, fermentation, host physiology, and performance. The enrichment of fibrolytic taxa under moderate CSB levels corresponds with increased NDF digestibility and higher concentrations of acetate and total VFAs, which are key metabolic fuels supporting higher growth rates [40]. These improvements in fermentative output help explain the enhanced ADG and feed efficiency observed in the LCSB and MCSB treatments. A Simultaneously, the reduction in pro-inflammatory cytokines and increased IgG suggest enhanced epithelial barrier stability and lowered state of immune activation. This likely reduced the nutrient and energy costs associated with inflammation, thereby permitting a greater allocation of resources toward growth. The reduction in ruminal lysozyme activity with CSB supplementation may further indicate diminished inflammatory pressure or epithelial challenge within the rumen. Although lysozyme activity was positively correlated with *Ruminococcus* abundance, this relationship reflects co-variation among individuals rather than a treatment-induced increase.

A particularly notable observation was the quadratic effect in rumen pH with CSB supplementation. High-concentrate diets commonly depress rumen pH, leading to microbial dysbiosis, reduced fibrolytic activity, and lower fermentation efficiency. CSB supplementation elevated rumen pH from 5.54 to 6.94, creating conditions more favorable for fibrolytic bacteria and enzymatic activity. While CSB contains buffer salts (NaHCO_3_ and NaH_2_PO_4_), the total intake of these salts is relatively low and unlikely to fully account for the observed magnitude of pH improvement [41]. Instead, we propose that the pH stabilization was largely mediated through microbial mechanisms. The CSB-induced shift toward a microbiota enriched with fibrolytic and potentially lactate-utilizing taxa (e.g., *Fibrobacter*, *Ruminococcus*, *unclassified Oscillospiraceae*) likely reduced lactate accumulation, thereby promoting a more stable and resilient rumen environment [42–44]. This microbial-mediated stabilization of fermentation explains the higher lipase and butyrate kinase activities observed in the MCSB group, as enzyme activity is highly sensitive to ruminal pH. These results underscore the synergistic roles of microbial community structure, fermentation dynamics, and enzymatic activity in mediating the physiological effects of CSB.

A consistent quadratic dose–response pattern was detected across growth performance, digestibility, fermentation, and microbial variables, with 5 g/kg emerging as the most effective supplementation level. This non-linear response can be interpreted through several interconnected physiological and ecological lenses. Several explanations may account for this non-linear response. First, from a microbial perspective, moderate levels of butyrate may optimally stimulate beneficial fibrolytic communities, while excessive doses could disrupt microbial equilibrium, fostering competitive inhibition or promoting less desirable microbial pathways [9,45]. Second, concerning host physiology, rumen epithelial uptake and metabolism of VFAs may approach saturation at higher butyrate loads, potentially leading to metabolic feedback inhibition that attenuates net benefits. Third, regarding the rumen environment, the beneficial buffering effect at moderate doses may give way to potential osmotic stress or disruption of fine-tuned acid-base regulation at excessively high salt (CSB) intakes. As this study did not include direct measurements of rumen epithelial morphology, transporter expression, or intracellular signaling, these proposed explanations remain mechanistic hypotheses rather than definitive evidence; therefore, further studies integrating rumen histological, molecular, and functional analyses are required to validate and elucidate the underlying mechanisms.

In summary, the findings demonstrate that CSB supplementation improved growth performance, nutrient utilization, immune status, fermentation patterns, and microbial structure in Gangba sheep. These coordinated responses suggest that CSB enhances digestive efficiency and overall physiological resilience under high-altitude feeding conditions. The consistent quadratic patterns observed across numerous parameters support the recommendation of 5 g/kg DM as an optimal supplementation level for practical production. Several limitations of this study must be acknowledged. First, while a sample size of six animals per group is acceptable for performance trials, the high inter-individual variability inherent to microbiota may limit the statistical power for detecting subtle microbial effects. Second, the study was conducted using a typical high-altitude breed (Gangba sheep) under a specific semi-controlled feeding system, which may affect the generalizability of the findings to other breeds or production systems. Third, testing only three discrete CSB doses may not fully delineate the complete dose-response curve. Finally, the lack of molecular and histopathological endpoints (e.g., rumen papillae morphology, tight junction proteins, inflammatory pathway markers) constrains deeper mechanistic interpretation. Future studies incorporating larger cohorts, a broader range of doses, diverse production environments, and integrated molecular analyses are warranted to validate and extend the present findings. Consequently, while a dosage of 5.0 g/kg DM is recommended for practical use in similar conditions, further validation in larger populations and diverse management settings is essential prior to large-scale application.

## CONCLUSION

The results of this study indicate that supplementation of 5.0 g/kg DM CSB under high-concentrate feeding conditions in Gangba sheep was associated with a more stable rumen pH, shifts in rumen microbial community structure, increased total VFAs, enhanced rumen enzyme activity, and improvements in several serum immune indicators. These responses were consistent with the observed increases in growth performance and feed efficiency. Overall, supplementation at this level may serve as a potential nutritional strategy to support productive performance, immune status, and rumen function in Gangba sheep. Further studies under diverse management and dietary conditions will be valuable for confirming these effects.

## Figures and Tables

**Table 1:** Basic diet formulation and nutrient levels

Feed ingredients	%	Nutritional level^1)^	%
Corn	60.0	Crude protein	10.36
Wheat	10.0	Ether extract	4.47
Soybean meal	5.0	Neutral detergent fiber	16.9
Barley straw	15.0	Acid detergent fiber	7.02
Bran	5.0	Ash	3.40
Hawthorn powder	2.0		
Soybean oil	1.5		
Premix^2)^	1.0		
NaCl	0.5		
Total	100		

1) Measure value.

2) Premix (content per kilogram): VA 700,000–1,000,000 IU, VE 4,000 IU, VK3 100 mg, VB1 100 mg, VB2 260 mg, VB12 1,000 μg, VB6 100 mg, VD3 200,000–500,000 IU, niacin 1,600 mg, pantothenic acid 500 mg, folic acid 50 mg, copper 0.2–1.5 g, iron 3–20 g, zinc 2–12 g, biotin 10 mg, manganese 2–14 g, iodine 10–100 mg, selenium 10–50 mg, cobalt 5–40 mg.

**Table 2:** Effects of chemically protected sodium butyrate on the growth performance of Gangba sheep

Items	Chemically protected sodium butyrate (g/kg DM basis)^1)^				SEM	p-values		
	
	CON	LCSB	MCSB	HCSB		Treatment	Linear	Quadratic
Initial body weight (kg)	19.66	19.55	19.63	19.28	0.48	0.9443	0.6308	0.8109
Final body weight (kg)	29.89^c^	32.39^ab^	33.04^a^	31.03^bc^	0.51	0.0014	0.0921	0.0003
Dry matter intake (DMI) (kg/d)	1.45^c^	1.50^b^	1.56^a^	1.54^ab^	0.02	0.0003	0.0002	0.0199
Average daily gain (ADG) (g/d)	170.56^c^	214.03^ab^	223.61^a^	195.69^b^	6.53	<0.0001	0.0087	<0.0001
Feed conversion ratio (FCR) (kg/kg)^2)^	8.54^a^	7.09^c^	6.99^c^	7.87^b^	0.27	0.0002	0.0471	<0.0001

1) CON: control group; LCSB: low-concentration chemically protected sodium butyrate diet (CON+1.0 g/kg CSB); MCSB: the medium-concentration chemically protected sodium butyrate diet (CON+5.0 g/kg CSB); HCSB: the high-concentration chemically protected sodium butyrate diet (CON+10.0 g/kg CSB).

2) FCR, calculated as DMI/ADG.

a,b Values within a row with no common superscripts differ significantly (p<0.05), n = 6.

DM, dry matter; SEM, standard error of the mean; Linear, linear effect; Quadratic, quadratic effect.

**Table 3:** Effects of chemically protected sodium butyrate on the apparent digestibility of nutrients of Gangba sheep

Items	Chemically protected sodium butyrate (g/kg DM basis)^1)^				SEM	p-values		
	
	CON	LCSB	MCSB	HCSB		Treatment	Linear	Quadratic
Crude protein (%)	65.47^b^	66.85^b^	70.92^a^	67.69^ab^	1.10	0.0157	0.0418	0.0495
Ether extract (%)	55.40	56.67	59.10	60.56	1.38	0.0643	0.0090	0.9450
Neutral detergent fiber (%)	69.27^b^	73.06^a^	75.35^a^	71.66^ab^	0.99	0.0029	0.0461	0.0012
Acid detergent fiber (%)	59.18	61.19	64.06	62.59	1.48	0.1483	0.0609	0.2516
DM (%)	66.58^b^	67.48^b^	70.55^a^	68.34^ab^	0.27	0.0002	0.0471	<0.0001
Organic matter (%)	51.93	53.26	55.15	55.80	1.45	0.2437	0.0493	0.8147

1) CON: control group; LCSB: low-concentration chemically protected sodium butyrate diet (CON+1.0 g/kg CSB); MCSB: the medium-concentration chemically protected sodium butyrate diet (CON+5.0 g/kg CSB); HCSB: the high-concentration chemically protected sodium butyrate diet (CON+10.0 g/kg CSB).

a,b Values within a row with no common superscripts differ significantly (p<0.05), n = 6.

DM, dry matter; SEM, standard error of the mean; Linear, linear effect; Quadratic, quadratic effect.

**Table 4:** Effects of chemically protected sodium butyrate on the serum immune and hormonal parameters of Gangba sheep

Items	Chemically protected sodium butyrate (g/kg DM basis)^1)^				SEM	p-values		
	
	CON	LCSB	MCSB	HCSB		Treatment	Linear	Quadratic
Immunoglobulin A (g/L)	1.27	1.45	1.35	1.22	0.08	0.2167	0.4642	0.0660
Immunoglobulin G (g/L)	11.54^b^	14.05^a^	13.15^ab^	13.83^a^	0.61	0.0374	0.0415	0.1533
Immunoglobulin M (g/L)	1.04	1.31	1.21	1.18	0.08	0.1287	0.3496	0.0688
Interleukin 2 (pg/mL)	33.72^a^	25.44^b^	27.31^b^	32.36^a^	1.67	0.0056	0.7685	0.0007
Interleukin 4 (ng/mL)	19.26^a^	14.04^b^	11.34^c^	11.62^c^	0.81	<0.0001	<0.0001	0.0028
Interleukin 6 (pg/mL)	74.82	74.74	72.78	72.87	2.29	0.8649	0.4552	0.9699
Tumor necrosis factor α (pg/mL)	37.32^a^	24.61^b^	18.75^b^	15.88^b^	3.73	0.0030	0.0004	0.2020
Interferon γ (pg/mL)	129.63	125.28	134.73	141.33	6.48	0.3587	0.1399	0.4082
Growth hormone (ng/mL)	7.37	8.05	8.09	7.93	0.64	0.8462	0.5561	0.5195
Growth hormone releasing hormone (pg/mL)	12.73^b^	15.97^a^	14.31^ab^	13.69^b^	0.59	0.0076	0.6470	0.0039
Insulin-like growth factor 1 (ng/mL)	230.53	221.51	216.22	226.28	13.34	0.8858	0.7653	0.4828
Myostatin (ng/mL)	38.81	42.66	41.50	38.96	2.25	0.5529	0.9448	0.1709

1) CON: control group; LCSB: low-concentration chemically protected sodium butyrate diet (CON+1.0 g/kg CSB); MCSB: the medium-concentration chemically protected sodium butyrate diet (CON+5.0 g/kg CSB); HCSB: the high-concentration chemically protected sodium butyrate diet (CON+10.0 g/kg CSB).

a–c Values within a row with no common superscripts differ significantly (p<0.05), n = 6.

DM, dry matter; SEM, standard error of the mean; Linear, linear effect; Quadratic, quadratic effect.

**Table 5:** Effects of chemically protected sodium butyrate on the rumen pH, ammonia nitrogen, volatile fatty acids (VFAs), and enzyme activities of Gangba sheep

Items	Chemically protected sodium butyrate (g/kg DM basis)^1)^				SEM	p-values		
	
	CON	LCSB	MCSB	HCSB		Treatment	Linear	Quadratic
pH	5.54^d^	6.14^c^	6.58^b^	6.94^a^	0.07	<0.0001	<0.0001	<0.0001
Ammonia nitrogen (mg/mL)	27.98^b^	55.74^a^	70.85^a^	58.32^a^	8.17	0.0103	0.0088	0.0228
Acetic acid (mmol/L)	44.78^b^	66.19^a^	69.62	64.94	4.42	0.0031	0.0128	0.0079
Propionic acid (mmol/L)	25.07	18.88	20.74	19.63	1.96	0.1461	0.1139	0.2093
iso-butyric acid (mmol/L)	1.17^bc^	1.57^a^	1.52^ab^	1.05^c^	0.13	0.0235	0.4857	0.0031
Butyric acid (mmol/L)	8.00^bc^	10.07^b^	16.12^a^	7.81^c^	0.75	0.0001	0.7956	<0.0001
Iso-valeric acid (mmol/L)	2.94	4.26	3.07	2.38	0.57	0.1618	0.2752	0.0942
Valeric acid (mmol/L)	1.78	1.28	1.15	0.98	0.28	0.2453	0.0580	0.5629
Total VFAs (mmol/L)	83.74^c^	102.58^b^	112.22^a^	93.79^b^	8.68	0.0230	0.0502	0.0015
Cellulase activity (μg/min/mL)	10.84	11.42	13.47	13.31	1.05	0.2229	0.0581	0.7252
Lysozyme activity (μg/mL)	24.95^a^	13.66^c^	8.01^d^	18.67^b^	3.69	0.0257	0.1534	0.0074
Neutral protease activity (nmol/min/mL)	3.82	5.59	4.78	4.59	1.93	0.9333	0.8635	0.6168
Lipase activity (U/mL)	1.05^b^	1.24^ab^	1.91^a^	2.24^a^	0.15	0.0083	0.0133	0.8405
α-Amylase activity (mg/min/L)	39.11	29.17	36.44	47.07	5.69	0.2015	0.2352	0.0856
Butyrate kinase activity (μg/min/L)	263.79^b^	257.59^b^	377.15^a^	289.62^b^	15.25	<0.0001	0.0024	0.1904

1) CON: control group; LCSB: low-concentration chemically protected sodium butyrate diet (CON+1.0 g/kg CSB); MCSB: the medium-concentration chemically protected sodium butyrate diet (CON+5.0 g/kg CSB); HCSB: the high-concentration chemically protected sodium butyrate diet (CON+10.0 g/kg CSB).

a–d Values within a row with no common superscripts differ significantly (p<0.05), n = 6.

DM, dry matter; SEM, standard error of the mean; Linear, linear effect; Quadratic, quadratic effect.

## Data Availability

The raw sequence reads from the rumen microorganisms were submitted to the NCBI Sequence Read Archive database, with the accession number PRJNA1280279.
